# The Book World of Medicine and Science

**Published:** 1902-08-09

**Authors:** 


### The Book World of Medicine and Science.

A Manual of Practical Medical Electricity, the Roentgen Rays and Finsen Light. By Dawson Turner, B.A., M.D., F.R C.P.Edin., M R C.P.Lond. Third Edition. (London: Baillière, Tindall, and Cox. 1902. Price 7s. 6d. net.)

This is a most useful and up to date manual; just what many a practitioner requires, since by a careful study of its pages he may place himself reasonably *en rapport* with much about which it is to be feared that many practitioners know far too little, and about which none can know too much. It begins at the very beginning, the first illustrations that meet the eye being pictures of bits of paper and pith balls attracted and repelled by excited glass rods, and other familiar elementary examples of electiical action. Then we come to an account of the different forms or manifestations of electricity and a really very full and completely illustrated description of the many forms of apparatus employed in their production and administration, this portion of the work ending with an account of the Tesla phenomena, "high frequency currents," and experiments with the ultra-violet rays. Part II. is devoted to electro-physiology, a very good account being given of the physiological effects resulting from the application of electricity, and of the differences between the actions of its several forms. A fairly full account is here given of the phenomena of electrolysis, and from the experiments described the reader is led to draw the conclusion that very many of the phenomena which are produced by the passage of electrical currents through the body are the outcome of interpolar chemical changes or rearrangement of ions, or of interpolar electrolytic action taking place wherever variations occur in the electrical conductivity of the tissues which form the electrolyte.

Part III. which deals with electro-diagnosis, brings us back to the bedside, and will be found full of useful information, while Parts IV. and V., which deal with electro-surgery and electro-therapeutics, may be accepted as trustworthy guides to the practical application of electricity in professional work. They serve well to show how much may be done in this way by anyone who takes the trouble to make himself familiar with the details of the various processes. As an example of the practical form in which the directions are given, we quote the following concerning the removal of superfluous hairs by electrolysis. "From four to six cells of an ordinary battery (5 m.a.) are required; an electrolysis epilation needle, with a make and break key on the handle, and a pair of epilation forceps. The patient should be comfortably seated or reclining in a good light. Place a medium-sized well-moistened pad-electrode attached to the positive pole over the sternum, and secure the cathode to the electrolysis needle. Grasping the hair in the epilation forceps, press the needle-point down for about an eighth of an inch into the hair follicle beside the hair. Make the current for a few seconds, a few bubbles of gas will escape, and the hair can be lightly drawn out. A stinging pain is felt during the passage of the current, but an anæsthetic is never required. About 10 to 15 hairs can be removed at a sitting. A 20 per cent. ointment of cocaine can be applied afterwards to soothe any irritation. A small red spot marks the site of each operation for a few days, but no scar results." The chapters on the Roentgen rays and the Finsen light are not so full in proportion to the attention which has lately been directed to these subjects as those in the more purely electrical portions of the book, but they are quite sufficient to give a good idea of what is being done in these departments, and of what is the *modus operandi* of the means employed. It is a most useful book.

The Elements of Mind. By H. Jamyn Brooks. (London: Longmans, Green and Co. 1902. Pp. 312. Price 10s. 6d. net.)

The author of this book believes that he has discovered "the Elements of Mind, which, when compounded with those of Force and Matter, contribute the mysterious substance we call Life." The whole work is founded on this thesis, and the various chapters are then produced and expounded as a scheme of psychology on a new basis. The line of reasoning taken is metaphysical and somewhat abstruse. The methods pursued are mainly deductive; there is little or no collecting and collating of data to build up conclusions such as the modern scientific method demands. Here, for example, is a specimen of the author's style taken from the opening chapter. "Whatever is elementary in nature must have real existence; every mental conception employs some elementary unanalysable entity. Hence every conception of the mind carries with it some real existence in nature; otherwise the conception would represent a *creation* of mind, which is inconceivable." Among other outcomes of this method of reasoning are a number of words and phrases such as the Greater Ego, the Greater Mind, the Greater Personality, and others used with great frequency but also with much looseness and confusion. In fact the chapter on the Ego and Greater Ego may be cited as a terrible example of confusion of ideas and looseness of reasoning put into a form that is barely intelligible. Then follow chapters on Consciousness, Reason, Will, Memory, Habit and Hypnotism, in all of which similar defects are apparent. In the concluding part of the work the author deals with physical forces, and seeks to prove that they too are substances, a chapter that would amuse physicists owing to its curious medley of errors and naïve ignorance of even the fundamental facts of physical science. The work is a grandiose and pretentious one, evidently written by one who has spent his leisure in metaphysical speculation of the dilettanti order and without a scientific groundwork in psychological or physical science. It is marked throughout by weakness of grasp, looseness of ideas, and a pseudo-scientific method which assumes much, proves little, and adds almost nothing to our stock of knowledge of the subject.

The Prevention of Infection in Public Vehicles. By Alfred Greenwood, M.D., Ch.B., D.P.H., L.R.C.P., L.R.C.S. (London: The Sanitary Publishing Company, Ltd. 1902. Pp. 65, Price 1s.)

The habit of indiscriminate spitting is in itself a disgusting one, but it is scarcely yet realised that it is also a source of danger to the community. All this is clearly stated in the useful little work before us, but the especial aim of the author is to draw attention to the dangers derived from germ-containing dust in public vehicles. It has been proved experimentally that the dust gathered in railway carriages may contain tubercle bacilli and in twelve samples collected two proved infectious when inoculated into animals. Dr. Greenwood calls attention to the curious anomaly in our sanitary laws that whereas vehicles for the transmission of animals must be cleansed and disinfected no such provisions apply to those used by people. He admits the difficulty of disinfecting the upholstery of railway compartments, and suggests that it should be fixed on detachable frames. But above all it is necessary to educate people not to spit, and where expectoration is unavoidable to carry in a special pocket some pieces of oiled paper and wrap up the sputum until it can be destroyed on reaching home, preferably by burning. The book is clearly and well arranged.

